# Utilising identifier error variation in linkage of large administrative data sources

**DOI:** 10.1186/s12874-017-0306-8

**Published:** 2017-02-07

**Authors:** Katie Harron, Gareth Hagger-Johnson, Ruth Gilbert, Harvey Goldstein

**Affiliations:** 1 London School of Hygiene and Tropical Medicine, 15-17 Tavistock Place, London, WC1 H 9SH UK; 2 Administrative Data Research Centre for England, UCL, 222 Euston Road, London, NW1 2DA UK; 3 Administrative Data Research Centre for England and UCL Great Ormond Street Institute of Child Health, 30 Guilford Street, London, WC1 N 1EH UK; 4 University of Bristol, Administrative Data Research Centre for England and UCL Great Ormond Street Institute of Child Health, 30 Guilford Street, London, WC1 N 1EH UK

**Keywords:** Data linkage, Record linkage, Administrative data, Linkage error, Linkage evaluation, Hospital admission

## Abstract

**Background:**

Linkage of administrative data sources often relies on probabilistic methods using a set of common identifiers (e.g. sex, date of birth, postcode). Variation in data quality on an individual or organisational level (e.g. by hospital) can result in clustering of identifier errors, violating the assumption of independence between identifiers required for traditional probabilistic match weight estimation. This potentially introduces selection bias to the resulting linked dataset. We aimed to measure variation in identifier error rates in a large English administrative data source (Hospital Episode Statistics; HES) and to incorporate this information into match weight calculation.

**Methods:**

We used 30,000 randomly selected HES hospital admissions records of patients aged 0–1, 5–6 and 18–19 years, for 2011/2012, linked via NHS number with data from the Personal Demographic Service (PDS; our gold-standard). We calculated identifier error rates for sex, date of birth and postcode and used multi-level logistic regression to investigate associations with individual-level attributes (age, ethnicity, and gender) and organisational variation. We then derived: i) weights incorporating dependence between identifiers; ii) attribute-specific weights (varying by age, ethnicity and gender); and iii) organisation-specific weights (by hospital). Results were compared with traditional match weights using a simulation study.

**Results:**

Identifier errors (where values disagreed in linked HES-PDS records) or missing values were found in 0.11% of records for sex and date of birth and in 53% of records for postcode. Identifier error rates differed significantly by age, ethnicity and sex (*p* < 0.0005). Errors were less frequent in males, in 5–6 year olds and 18–19 year olds compared with infants, and were lowest for the Asian ethic group. A simulation study demonstrated that substantial bias was introduced into estimated readmission rates in the presence of identifier errors. Attribute- and organisational-specific weights reduced this bias compared with weights estimated using traditional probabilistic matching algorithms.

**Conclusions:**

We provide empirical evidence on variation in rates of identifier error in a widely-used administrative data source and propose a new method for deriving match weights that incorporates additional data attributes. Our results demonstrate that incorporating information on variation by individual-level characteristics can help to reduce bias due to linkage error.

## Background

Linkage of administrative data is an important tool for service evaluation and research, as individual-level information can be combined in a relatively cost-effective and timely manner compared with conventional data collection models. Most administrative data sources were not developed with linkage in mind, posing unique challenges for identifying the same individual in different sources [1]. Typographical errors, missing values and identifiers that change over time can prevent records from matching and lead to linkage error (false-matches and missed-matches) [2, 3]. Even low error rates can lead to biased results, particularly when records from particular types of individuals or organisations are less likely to link successfully than others. Such ‘differential’ linkage can lead to a form of bias in analysis, for example when specific groups of records are misclassified or excluded from the linked dataset [4–8].

For linkage of data sources that do not contain a reliable unique identifier, probabilistic methods are commonly used [9, 10]. Probabilistic linkage makes use of variables such as sex, date of birth and postcode to create a match weight for classifying records as matches or non-matches. Match weights are traditionally based on the Fellegi-Sunter approach using conditional probabilities derived from estimated rates of errors in identifiers: the probability that identifiers agree given records belong to the same subject (*m*-probability), and the probability that identifiers agree given records belong to different subjects (*u*-probability) [11, 12]. Conditional probabilities can be derived from ‘training’ data, i.e. a sub-sample of data where the true match status for each record pair is known (supervised matching) [13]. If no training data are available, probabilities are typically estimated using statistical methods, such as the expectation-maximisation (EM) algorithm [14].

There are several problems associated with the calculation of probabilistic match weights using the traditional approach. Firstly, match weights are calculated assuming that identifier errors occur randomly within a dataset, and that the probability of an identifier error is unrelated to any other characteristic (age, ethnicity etc.) [11]. However, this assumption is often invalid: data quality is often associated with individual-level characteristics and can also vary on an organisational level [15]. These associations are typically ignored, unless these characteristics are incorporated into a blocking scheme with match weights being produced separately for each block. Secondly, match weights are typically calculated by summing the logarithms of m- and u-probability ratios across identifiers. This requires the assumption that identifier errors are independent (i.e. agreement on year of birth is independent of agreement on forename) - an assumption that often fails and can lead to misclassification of record pairs [16].

One approach to overcome these problems is to estimate match weights jointly over a set of identifiers (the agreement pattern), thus overcoming the need for independence between identifiers. It is also possible to calculate match weights allowing dependence on individual- and/or organisational-level covariates. Although characteristics of the identifying variables, such as the frequency of common or rare surnames, are often incorporated into match weight calculation, this has not been the case for individual characteristics that are not used for matching (e.g. ethnicity) or at an organisational level (e.g. by hospital). The present study aims first to provide empirical evidence on the associations between identifier error rates and individual characteristics in a national administrative data source (Hospital Episode Statistics; HES). Quality of identifier recording in HES is likely to be representative of other administrative sources, i.e. those where identifiers are input using a range of IT systems, and so information on identifier error rates will be relevant to linkage of other large administrative data sources. Secondly, we develop methods to estimate match weights without relying on the independence assumption, and incorporating individual or organisational-level attributes, and evaluate these weights as alternatives to traditional probabilistic match weights.

## Methods

### Data

Hospital Episode Statistics (HES) is an administrative data source containing information on all admissions to NHS hospitals in England. Linkage of HES is coordinated through NHS Digital (previously known as the Health and Social Care Information Centre) [17]. The HES extract used for this study had previously been linked with a reference (gold-standard) dataset of records extracted from the Personal Demographic Service (PDS), which is also coordinated by NHS Digital (http://systems.digital.nhs.uk/demographics/pds). PDS contains the latest demographic details corresponding to a given NHS number. PDS also contains historical information such as previous addresses and is used for the NHS number tracing service (known as the Demographics Batch Service) and to provide identifiers for the NHS Patient Spine. Linkage with PDS reference data allowed us to quantify identifier errors. In this study, we define identifier error as discrepancies between PDS and HES, e.g. where identifiers had been recorded incorrectly, had legitimately changed over time (e.g. postcode) or were missing in HES.

For the purposes of this study, we defined our true (reference) match status by agreement or disagreement of NHS number between HES and PDS. We used a random sample of 10,000 record pairs from HES inpatient data linked with PDS, for the financial year 1^st^ April 2011 to 31^st^ March 2012, for each of three cohorts defined by date of birth: i) infants aged <1 year; ii) children aged 5–6 years; and iii) young adults aged 18–19 years. For each age cohort, the set of matches was created by identifying the PDS record associated with the NHS number on each HES record (*n* = 10,000 matches). The set of non-matches was created by identifying all PDS records with different NHS numbers to each HES record. This resulted in (10,000 × 10,000)-10,000 = 99,000,000 non-matches for each age cohort. However, the majority of these non-matches did not agree on any identifier, or only agreed on sex, and so were excluded from consideration. This resulted in around 30,000 non-matches for each age cohort.

The data used for this study comprised patterns of agreement/disagreement between date of birth, sex and postcode in HES-PDS linked pairs, but contained no actual identifiers. Agreement patterns were aggregated by age cohort, sex and ethnic group.

### Identifier error rates

We estimated identifier error rates for sex, date of birth and postcode, based on the number of times these identifiers disagreed in matched HES-PDS records. We modelled the risk of identifier error using logistic regression with a set of attribute predictors recorded in HES (ethnicity, age and sex). We used a multi-level model with hospital as a random effect to explore organisational-level variation. Dependence between pairwise identifiers was also tested using multi-level logistic regression models using Stata [18].

### Probabilistic match weights

1. Traditional probabilistic match weights (assuming independence between identifiers)
  We derived conditional probabilities for sex, date of birth and postcode based on the observed error rates for each identifier. Probabilities were derived from the number of times an identifier agreed or disagreed in pairs of matched HES-PDS records, e.g. for sex:
  $$ \begin{array}{l} m- probability = {m}_{sex}= P\left( agree\  on\  sex\Big| M\right)\hfill \\ {} u- probability = {u}_{sex} = P\left( agree\  on\  sex\Big| U\right)\hfill \end{array} $$
  where M represents a match and U represents a non-match. Missing values were treated as disagreement.
  Match weights were then derived by summing the log-ratio of m- and u-probabilities over all *k* identifiers, i.e.
  $$ \boldsymbol{W} = {\displaystyle \sum_k} l o{g}_2\left(\frac{m_k}{u_k}\right) = l o{g}_2\left(\frac{m_{sex}}{u_{sex}}\right)+ l o{g}_2\left(\frac{m_{dob}}{u_{dob}}\right)+ l o{g}_2\left(\frac{m_{postcode}}{u_{postcode}}\right) $$
2. Match weights incorporating dependence between identifiers
  Each HES-PDS record pair was associated with an agreement pattern *φ* representing agreement or disagreement on the joint set of three identifiers {sex, date of birth, postcode}. For binary agreement (agree = 1; disagree = 0), there are 2^3^ = 8 possible agreement patterns for sex, date of birth and postcode: {1,1,1}, {1,1,0} … and {0,0,0} etc. Conditional probabilities were derived jointly over all identifiers for each observed agreement pattern, e.g. for agreement on sex, date of birth and disagreement on postcode, represented as {110}:
  $$ \begin{array}{l} m- Probability={m}_{\varphi}= P\left( agree\  on\  sex\  and\  date\  of\  birth,\  disagreement\  on\  postcode\ \left| M\right.\right)= P\left(\varphi =\left\{110\right\}\ \left| M\right.\right)\hfill \\ {} u- probability={u}_{\varphi}= P\left( agree\; on\; sex\  and\  date\  of\  birth,\  disagreement\  on\  postcode\ \left| U\right.\right)= P\left(\varphi =\left\{110\right\}\ \left| U\right.\right)\hfill \end{array} $$
  Match weights were then derived as:
  $$ W={ \log}_2\left(\frac{m_{\varphi}}{u_{\varphi}}\right) $$
3. Attribute-specific and organisational-specific match weights
  We derived attribute-specific match weights using the procedures described above, but now for each combination of characteristics as recorded in PDS (age cohort, sex, ethnic group, N combinations = 36). This process is distinct from blocking, in that agreement on any of these attributes is not required for linkage (and attribute-specific weights can be calculated for variables not used within the linkage, e.g. ethnic group). Organisational-specific match weights were derived by calculating m- and u-probabilities separately for each hospital (N hospitals= 388). Attribute-specific and organisational-specific match weights were calculated in the traditional manner (i.e. assuming independence between identifiers), as it was not possible to stratify each agreement pattern by age, sex, ethnicity due to low numbers.

### Simulation study

#### Aim

We performed a simulation study to determine the effect of the identifier-independence assumption and the value of incorporating attribute information into match weight calculation. Our scenario was linkage of hospital admissions records containing sex, date of birth, postcode, and NHS number. The aim was to estimate readmission rates by linking multiple hospital records for the same individual over time. Where there was a match between hospital records, this indicated that an individual had been admitted multiple times within the study period. Individuals with only a single hospital record and no matches were admitted only once during the study year.

#### Data generating mechanism

For each simulation, we created our ‘matches’ by randomly sampling agreement patterns (with replacement) from matched pairs in the HES-PDS extract, retaining distributions of age, sex and ethnicity from the original data. We created our ‘non-matches’ by sampling agreement patterns from non-matches in the HES-PDS extract. Sampling of matches and non-matches was stratified by age, sex and ethnicity, in order to reflect differences in readmission rates observed in the literature.[19] This approach avoided any distributional assumptions about identifier error rates for date of birth, sex or postcode, and also preserved associations between identifiers and individual characteristics.

Since by design, the original HES-PDS extract only included records that agreed on NHS number, we introduced NHS number identifier error rates representative of those observed in the literature [20, 21]. We used several scenarios to determine the effect of different NHS number error rates on results:

1. NHS number was randomly missing or incorrect in 30% of records
2. NHS number was randomly missing or incorrect in 0.5% of records.
3. NHS number was missing or incorrect in 30% of records overall, but was twice as likely to contain errors if there were errors in any of the other identifiers (sex, date of birth or postcode).
4. NHS number was missing or incorrect in 30% of records overall, but errors were distributed with the same pattern as errors in ethnicity (as observed in the HES-PDS extract).

For each simulation, records were rank ordered by match weight, and a cut-off threshold for classifying records as matches was chosen by determining the maximum weight or probability that would not exceed a false-match rate of 1% (or 99% specificity). It was possible to fix this threshold since the true match status was known in the simulated data, although this would not be possible in real data.

#### Comparisons

Results from three approaches were averaged over 500 simulated datasets and compared with those from traditional match weights: i) match weights incorporating dependence between identifiers (based on agreement patterns), ii) attribute-specific match weights (based on 36 different combinations of characteristics) and iii) organisational-specific match weights (based on 388 hospitals). We compared sensitivity (i.e. the proportion of true matches that were identified) between methods and compared estimated readmission rates from each method with the ‘true’ readmission rate within 12 months (8.8%) in the simulated data. We assessed the performance of each method by measuring bias, i.e. the percentage difference between estimated and true readmission rates.

## Results

### Identifier error rates

Identifier errors (including missing values) were found in 0.11% of records for sex and date of birth, and in 53% of records for postcode. In these data, there was no evidence of dependence between postcode and date of birth or sex (*p* = 0.266 and 0.187 respectively from the multi-level logistic regression model). Although the error rate for date of birth was low, errors in this variable were more likely to occur in records where there was also an error in sex (*p* = 0.021).

**Fig. 1:**
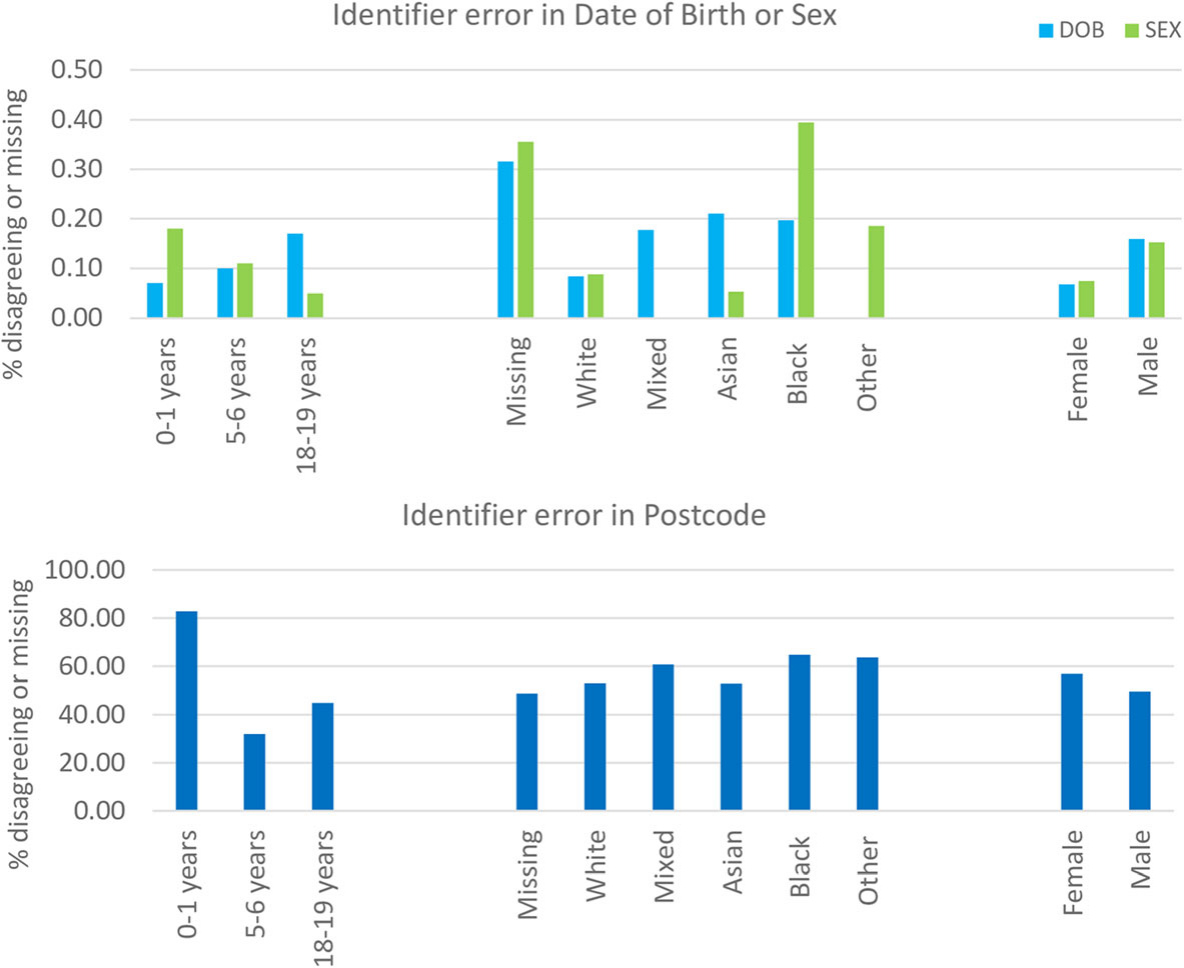
Percentage of HES-PDS linked records with disagreeing or missing identifiers according to age, ethnicity and sex. The larger identifier error rates in postcode reflect that postcode was missing for 83% of records for infants aged 0–1 years

The probability of identifier error (disagreement of identifier values between HES and PDS) differed significantly according to age (*p* < 0.0001), ethnicity (*p* = 0.0005) and sex (*p* < 0.0001) (Fig. 1). Identifier errors occurred less frequently in records from females compared with males (odds ratio 0.84; 95% CI 0.81-0.86); and were lowest for Asian ethnicity (odds ratio 0.89; 95% CI 0.84-0.94 compared with White ethnicity). Across all identifiers, errors occurred less frequently in 5–6 year olds and 18–19 year olds compared with infants (odds ratios 0.39; 95% CI 0.37-0.40 and 0.37; 95% CI 0.36-0.39 respectively). However, patterns differed according to the identifier: sex was more likely to be correct in 18–19 year olds than infants, but the pattern was reversed for date of birth (Fig. 1).

**Fig. 2:**
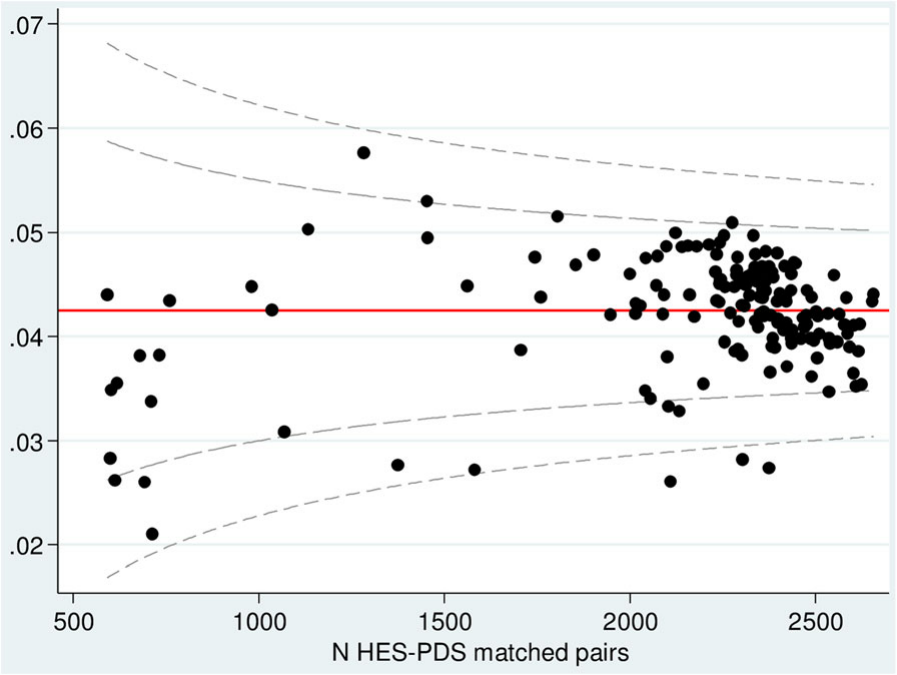
Variation in identifier error rates by hospital provider (*n* = 167). Each dot represents one hospital (hospitals with <500 matches were excluded). Inner lines = 95% control limits; outer lines = 99.8% control limits

Multi-level logistic regression showed there was substantial variation on an organisational level although no particular hospital provider had a significantly higher error rate than the overall mean (Fig. 2).

### Probabilistic match weights

Absolute values differed for traditional match weights, match weights incorporating dependence between identifiers, and attribute-specific match weights (Table 1). The ordering of weights (and therefore of record pairs) was the same using both traditional weights and weights incorporating dependence. However, for attribute-specific weights, ordering differed according to individual characteristics. For example, for 0–1 year olds, agreement on date of birth and sex only had a higher weight than agreement on sex and postcode only, but these weights were reversed for the older age groups. Variation in attribute-specific match weights reflected underlying identifier error rates. For example, the match weight for agreement on date of birth and sex but disagreement on postcode was 9.2 for infants, but 7.9 for 5–6 year olds, reflecting the fact that postcode was more likely to be missing in infant records.

**Table 1:** Traditional match weights, match weights incorporating dependence between identifiers, and attribute-specific match weights according to agreement pattern {date of birth, sex, postcode}. Record pairs with no agreement on any identifiers, or where only sex agreed (agreement patterns {000} and {010}), were assumed to be non-matches and excluded

		Agreement pattern {date of birth, sex, postcode}					
		001	100	011	101	110	111
N Matches		1	21	18	12	15,924	14,009
N Non-matches		259	414,307	248	4	415,888	10
Match probability^a^		0.0039	0.0001	0.0726	0.7500	0.0369	0.9993
Traditional match weight		5.3	−1.0	9.6	14.9	8.6	23.7
Match weight assuming dependence		−0.5	−1.2	9.3	17.8	8.6	27.6
Attribute-specific match weight:							
Sex	Female	−1.7	−1.7	8.7	17.3	8.7	27.7
	Male	0.4	−0.9	9.7	18.1	8.5	27.5
Age	0–1 years	−0.5	0.1	8.6	18.5	9.2	27.6
	5–6 years	−0.2	−2.0	9.6	18.2	7.9	28.0
	18–19 years	−1.5	−2.6	9.5	16.2	8.3	27.2
Ethnicity	Missing	2.7	0.3	10.8	19.5	8.5	27.6
	White	−1.3	−1.6	8.8	17.4	8.6	27.6
	Mixed	1.8	−0.4	10.9	19.4	8.8	28.6
	Asian	−0.4	−2.3	10.4	16.9	8.6	27.8
	Black	1.6	0.9	9.6	19.1	8.9	27.1
	Other	1.4	−0.2	10.5	19.0	8.8	28.1
Organisational-specific match weight (mean)		5.7	1.3	12.3	20.7	8.1	25.4

^a^Match probability = N matches/Total record pairs

### Simulation study

Sensitivity of linkage varied from 79% using traditional match weights and match weights incorporating dependence, to 97% using attribute-specific match rates. With an error rate of 30% in NHS number, all methods underestimated the ‘true’ overall readmission rate of 8.8%, except for the organisation-specific match weights (Table 2). Traditional match weights and match weights incorporating dependence provided similar results; organisational- and attribute-specific match weights performed best overall.

**Table 2:** Simulation study results: estimated readmission rates. The ‘true’ readmission rate was 8.8%

		NHS number error distribution in simulated data			
		30%, random	0.5%, random	30%, associated with other identifier errors	30%, associated with ethnicity
Traditional match weight	% readmitted	7.4	7.4	6.9	7.4
	Standard error	0.002	0.002	0.002	0.002
	% bias	−15.9	−15.9	−21.3	−15.7
Match weight incorporating dependence	% readmitted	7.4	7.4	6.9	7.4
	Standard error	0.002	0.002	0.002	0.002
	% bias	−16.0	−16.0	−21.4	−15.8
Attribute-specific match weight	% readmitted	8.7	8.7	8.7	8.8
	Standard error	0.002	0.002	0.002	0.002
	% bias	−0.6	−0.6	−0.9	−0.2
Organisation-specific match weight	% readmitted	8.8	8.8	8.8	8.8
	Standard error	0.002	0.002	0.002	0.002
	% bias	0.2	0.2	−0.1	0.2

Bias in estimated readmission rates was highest when NHS number errors were more likely to occur in records with at least one other identifier error (21% bias using traditional weights or weights incorporating dependence, 3% using attribute-specific weights, 0.1% using organisational-specific weights).

**Fig. 3:**
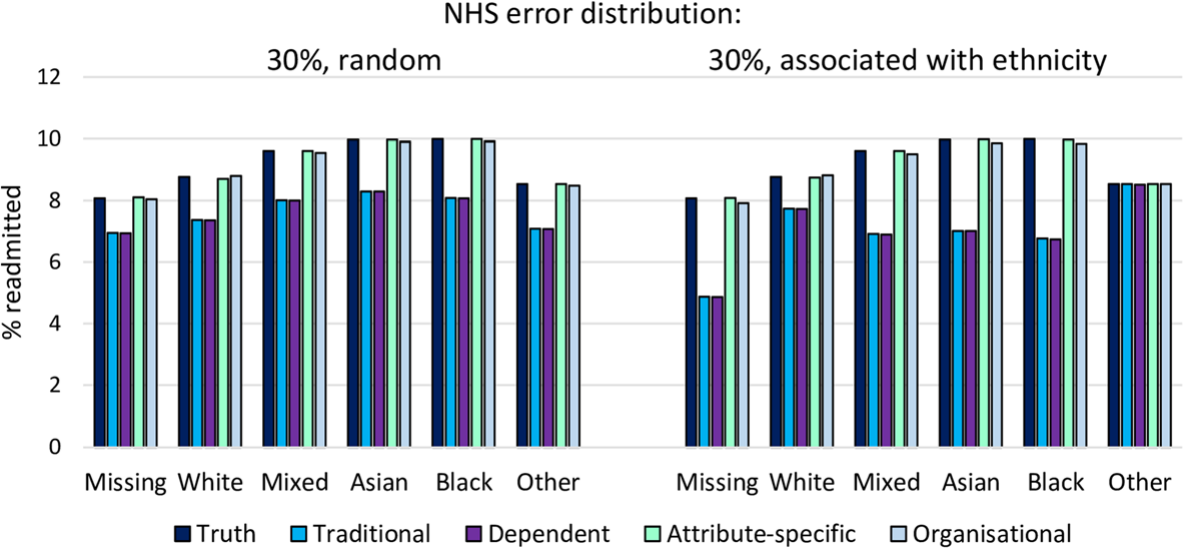
Simulation study results: estimated readmission rates by ethnicity, according to NHS number error rate distribution

**Fig. 4:**
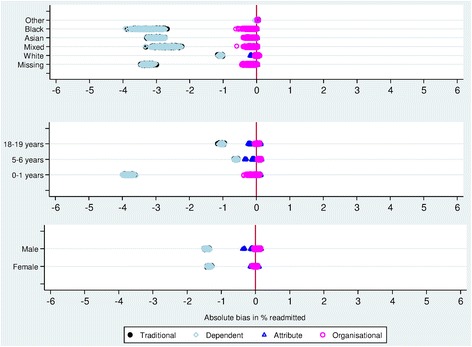
Simulation study results: absolute bias in estimated readmission rates for NHS number error associated with ethnicity. Results for traditional match weights fall behind those for weights incorporating dependence between identifiers

Errors in date of birth were highest in records with missing ethnicity, lower in the White group compared to Mixed, Asian, or Black, and there were no errors in the ‘Other’ category (Fig. 1). When this distribution was applied to NHS number errors, bias varied accordingly: little bias was introduced to the estimated readmission rate for the ‘Other’ group, but estimates for the Missing group were substantially biased (Figs. 3–4). Attribute- or organisational-specific weights performed well at handling these dependencies, with an overall bias of 1% and 0.2% respectively,

## Discussion

Our study provides empirical evidence on variation in identifier error rates by individual characteristics in a widely-used and extensive administrative data source. This information will be valuable for other researchers assessing the feasibility of linkage with administrative data sources, particularly where no training data are available, as the identifier error rates observed in HES will provide an appropriate starting point for estimating m- and u-probabilities in other similar datasets. We provide methods for incorporating dependence between identifiers, and variation in identifier errors by individual and organisational-level characteristics, into match weight calculation. Our simulation study demonstrated that match weights incorporating individual characteristics or organisational variation were effective at reducing bias associated with errors in linkage, particularly when errors are distributed non-randomly.

Results from our simulation study support a large body of literature showing that substantial bias can be introduced into results of analyses based on data containing linkage errors [22–25]. This is particularly important when error is non-random, i.e. dependent on individual-level characteristics, and when there are a large number of missed-matches (e.g. with linkage requiring exact matching of identifiers). Evidence from previous studies highlights that the most vulnerable groups are those most likely to be affected by linkage error [2, 26]. In our study, readmission rates estimated using linkage with traditional match weights were underestimated, due to low sensitivity when fixing the false-match rate at 1%. In practice, false-match rates are often lower than 1%, corresponding with a lower sensitivity (where false-matches are avoided, to the expense of missed-matches) and a greater risk of under-estimated readmission rates [15, 27]. Bias was greatest for Mixed, Asian, or Black ethnic groups, meaning that relative comparisons by ethnicity would be biased using match weights derived by the traditional method. However, we show that attribute- or organisational-specific match weights, incorporating information on variation in identifier errors, can substantially reduce bias associated with linkage error. Additional methods for handling linkage error, such as incorporating match weights into analysis in a multiple imputation framework, could be used to reduce bias further [23, 28].

Incorporating information on individual or organisational characteristics, or dependence between identifiers, into match weight estimation is a relatively simple process, given a large enough sample from which to estimate the relevant parameters. In practice, detailed information on identifier error rates is not always available and parameters are often derived from a sample of data. Where a large enough sample on which to base estimates of error rates is not available, it would be possible to incorporate characteristics into latent class models such as the Expectation-Maximisation (EM) algorithm, which can be used to estimate conditional probabilities for the traditional Fellegi-Sunter approach [14, 29]. The value of incorporating information on record attributes is likely to be most evident in linkage of large-scale administrative datasets, particularly where records are grouped, for example by organisation or region. However, our study used a relatively simple design of linkage within one longitudinal dataset, and further evaluation is required to understand performance and practicalities of the method in large, complex linkages involving multiple files.

There is limited evidence on how the failure of the assumption of independence affects linkage quality over and above the calculation of match weights. Tromp et al. (2008) found that dependence between highly correlated identifiers (such as expected birth and actual date of birth) had a negative impact on match weights and that this resulted in an incorrect ranking of record pairs ordered by match weight [16]. Similarly, Herzog et al. (2010) found that match weights assigned to non-dependent identifiers were too low in the presence of dependent identifiers [30]. Methods for accounting for dependence between identifiers have also been shown to improve the quality of linkage [31]. Others believe that the impact of dependence between identifiers is small, and that the failure of the independence assumption can be ignored [32, 33]. In our study, ordering of record pairs based on weights incorporating dependence between identifiers was the same as with traditional match weights, mainly due to a lack of strong dependence between errors in sex, postcode and date of birth observed in HES. However, incorporating dependence into match weights may become more important in data where obvious dependencies do exist, although handling dependence between a large number of identifiers may become impractical.

A major strength of this study was the use of a large, generalizable administrative data source that is frequently linked with other datasets and used for commissioning and monitoring of the NHS in England and for research. Our study demonstrates the usefulness of the PDS as a reference dataset. There is a lack of published information available on PDS but it holds potential for developing a better understanding of the mechanisms underlying identifier errors, for improving data linkage methods, and for validating identifiers in HES [34]. However, our study was limited by the assumption that agreement on NHS number between HES and PDS indicated that records belonged to the same individual. In reality, NHS number is not always a reliable identifier for linkage [20]. If well-completed NHS number is indicative of good data quality more generally, we may have underestimated identifier error rates through our study design. In addition, we based our extract on date of birth, and so excluded all records where date of birth was missing. We also used a one year study period, and therefore would not have captured changes in postcode over time. Inspection of PDS reveals that 55% of children have at least two postcodes in their first year of life and 69% have at least two postcodes by age 5/6 (19% have four or more different postcodes by this age). In our simulation study, we fixed our threshold at a false-match rate of 1%. In practice, choice of appropriate thresholds can be difficult, and is typically chosen based on a sample of manually-reviewed records, or using synthetic data [35].

## Conclusions

Incorporating information on individual characteristics or organisational variation into match weight calculation can reduce bias associated with errors in linkage, particularly when errors are distributed non-randomly. Continued improvement of linkage methods will allow more efficient exploitation of administrative data sources, reduce bias associated with linkage of imperfect identifiers and improve the reliability and transparency of analysis based on linked data. This will improve the ability of those working in government and health policy, who frequently use research data generated from administrative data sources to inform health policy, to make informed decisions on patient care and health systems. Evaluation of services for specific age or ethnic groups can be important for policy, but as our study shows, results for specific groups can be biased if associated linkage error is not addressed. Careful consideration should be given to the trade-off between bespoke linkage strategies for each study (that prioritise the quality of linkage) versus routine linkage systems that maximise efficiency and security. In order for data users to understand the limitations of linked data sources, it is vital that information on linkage quality and error rates are made available on release of linked data. Data providers need to improve transparency about data processing before during and after linkage.

## Abbreviations

HES: Hospital Episode Statistics
NHS: National Health Service
PDS: Personal Demographic Service
